# Diaphragmatic Hernia

**Published:** 1878-06

**Authors:** 


Diaphragmatic Hernia.—N. Hensley, æt. 45. About the last of October, 1876, was stabbed in the left side, between the sixth and seventh ribs, about four inches below and behind the left nipple. Was called to see him with Dr. Clemments, a few minutes after it happened. We probed the wound enough to be certain it had penetrated the thoracic cavity. Had him placed with the left side down. There was considerable hemorrhage. He was kept very quiet four or five days, when he was removed to his home, four miles distant. In three weeks he was able to come to town. I saw and prescribed for him frequently in the next twelve months. His health was not good, though he was able most of the time to do the work of a farm hand. Complained very much of pain in his left shoulder, ranging down through the precordial region to the epigastrium. A gurgling sound was discovered by auscultation, and a clicking sound of the heart. There was also intermission of the pulse. In March, 1877, he had a severe attack simultating gastritis, with obstinate constipation, which resisted for four days the usual means to remove it.

When the bowels were freely opened, immediate relief was obtained. Nov. 10, 1877, worked hard all day, digging potatoes; ate a hearty supper; soon after eating was taken with a violent pain in the left shoulder, extending down to the epigastrium, and incessant vomiting. Threw up everything as soon as swallowed. The bowels remained obstinately constipated, and nothing gave even temporary relief, except hypodermic injection of morphia. He died on the 18th. A *post-mortem* examination was held—present, Dr. Thomas Linley and Dr. C. H. Linley, of Salem, Kentucky.

Rigor mortis well marked, body emaciated, no distension of the abdomen except a slight fullness at the epigastrium. On removing the sternum, a large, mahogany colored tumor, which proved to be the fundus of the stomach, occupied and nearly filled the left side of the thorax, the heart being crowded over to the right side beyond the median line. The left lung greatly compressed and hepatized. The stomach, or rather the strangulated portion above the diaphragm, was distended with gas, but contained no fluid or other ingesta. A part of the omentum and transverse colon were also included in the hernial opening, which was about one and a half inches in diameter, and presented a ragged border, and corresponded with a mell-marked cicatrice where the knife entered between the sixth and seventh ribs.

The parts embraced in the hernial opening appeared to be gangrenous, and recent adhesions of the bowels immediately below the seat of the stricture were noticed. The small intestines were empty, and all the abdominal viscera, except in the nigh neighborhood of the strangulation, appeared to be healthy.

